# Successful Erlotinib Treatment for a Patient with Gefitinib-Related Hepatotoxicity and Lung Adenocarcinoma Refractory to Intermittently Administered Gefitinib

**DOI:** 10.1155/2011/812972

**Published:** 2011-12-25

**Authors:** Tatsuya Nagano, Yoshikazu Kotani, Kazuyuki Kobayashi, Masahiro Katsurada, Yukihisa Hatakeyama, Suya Hori, Daisuke Tamura, Daisuke Kasai, Yasuhiro Funada, Yoshihiro Nishimura

**Affiliations:** Division of Respiratory Medicine, Department of Internal Medicine, Kobe University Graduate School of Medicine, 7-5-1 Kusunokicho, Chuo-ku, Kobe, Hyogo 650-0017, Japan

## Abstract

A 73-year-old Japanese man was histologically diagnosed with lung adenocarcinoma harboring an exon 19 deletion in the epidermal growth factor receptor. The patient was treated with gefitinib for 6 weeks until he developed substantially elevated hepatic enzyme levels that resulted in the discontinuation of gefitinib. Gefitinib was reintroduced with an intermittent treatment schedule after the transaminase levels normalized, but the patient's enzyme levels rose again, and the cancer progressed. Gefitinib was eventually replaced with erlotinib. There was stable disease for 7 weeks without any signs of liver toxicity. Thus, erlotinib may be a beneficial and well-tolerated treatment option for patients with gefitinib-related hepatotoxicity.

## 1. Introduction

Recent reports suggest that epidermal growth factor receptor tyrosine kinase inhibitors (EGFR-TKIs) induce an early and dramatic response in patients with non-small-cell lung cancer (NSCLC) [1]. Subgroup analyses revealed that responsiveness to EGFR-TKI is more frequently observed in female nonsmokers with adenocarcinoma and that responsiveness is associated with specific gain-of-function mutations at exons 18, 19, and 21 of the EGFR gene, which are found in 10% of NSCLC cases in Europe and the United States and in 26% of NSCLC cases in East Asia [2].

Gefitinib is generally reported to be well tolerated, and the most common side effects consist of mild to moderate gastrointestinal disturbances and skin reactions [3–6]. Most patients had no deterioration in hepatic function during the trial, and occurrences of elevated levels of transaminases were generally grade 1, as assessed by the National Cancer Institute Common Toxicity Criteria version 2.0 grading system, and asymptomatic [3]. However, gefitinib-related hepatotoxicity was a dose-limiting toxicity in phase 1 studies [7–10]. Recently, the incidence of grade 3/4 liver transaminase levels, as assessed by the Common Terminology Criteria for Adverse Events (CTCAEs version 3.0) grading system proposed by the National Cancer Institute, was reported to be significantly higher with gefitinib than with carboplatin plus paclitaxel in a phase 3 study (9.4% versus 1.0%, resp.) [5]. Furthermore, in the Iressa Dose Evaluation in Advanced Lung Cancer (IDEAL 1) trial, 2% of patients receiving gefitinib alone at a dose of 250 mg once daily developed grade 3 or 4 elevations of hepatic enzymes that necessitated the cessation of treatment [3]. Therefore, an effective treatment method needs to be developed for patients for whom gefitinib has been discontinued because of severe hepatotoxicity irrespective of their response to gefitinib.

The present study describes the case of a 73-year-old Japanese man who was histologically diagnosed with lung adenocarcinoma harboring an exon 19 deletion in the epidermal growth factor receptor and who developed a substantial elevation of hepatic enzymes during gefitinib treatment. Following the case presentation, we discuss the treatment strategy for gefitinib-related hepatotoxicity through a review of pertinent reports.

## 2. Case Presentation

A 73-year-old Japanese man with a 17 pack-year smoking history was diagnosed with lung adenocarcinoma and underwent a right lower lobectomy in February 2004. The lung adenocarcinoma was pathologically characterized as T2N1M0, stage IIB, according to the TNM classification of the International Union Against Cancer (UICC) [11]. Twenty months later, the tumor had relapsed in the pleura and lymph node, but the relapse was asymptomatic, so the patient was routinely monitored. In February 2008, the patient developed bone and brain metastasis, and he received gamma knife radiation and first-line chemotherapy with carboplatin (area under the plasma concentration time curve (AUC) of 5, day 1) and gemcitabine (1000 mg/m^2^, days 1 and 8). However, this chemotherapy was discontinued after two cycles due to the continued development of tumor. The patient developed a lumbar backache, and magnetic resonance imaging scans of the thoracolumbar vertebrae showed bone metastases at the fourth and tenth thoracic vertebrae. He underwent radiation therapy (30 Gy/10 Fr) for the bone metastases and subsequently received second-line treatment with gefitinib (250 mg/day) in December 2008, as mutation analysis of a lung cancer specimen obtained from the right lower lobectomy detected the presence of an exon 19 deletion in EGFR. The tumors exhibited some reduction in size, and the patient had radiological stable disease, neither a partial response nor progressive disease, according to the Response Evaluation Criteria in Solid Tumours (RECIST).

**Figure 1:**
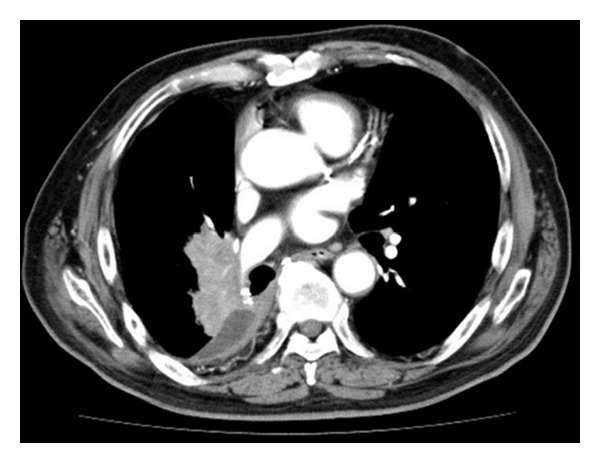
Computed tomography (CT) scan of the chest shows a mass opacity involving the right hilar lymph node.

**Figure 2:**
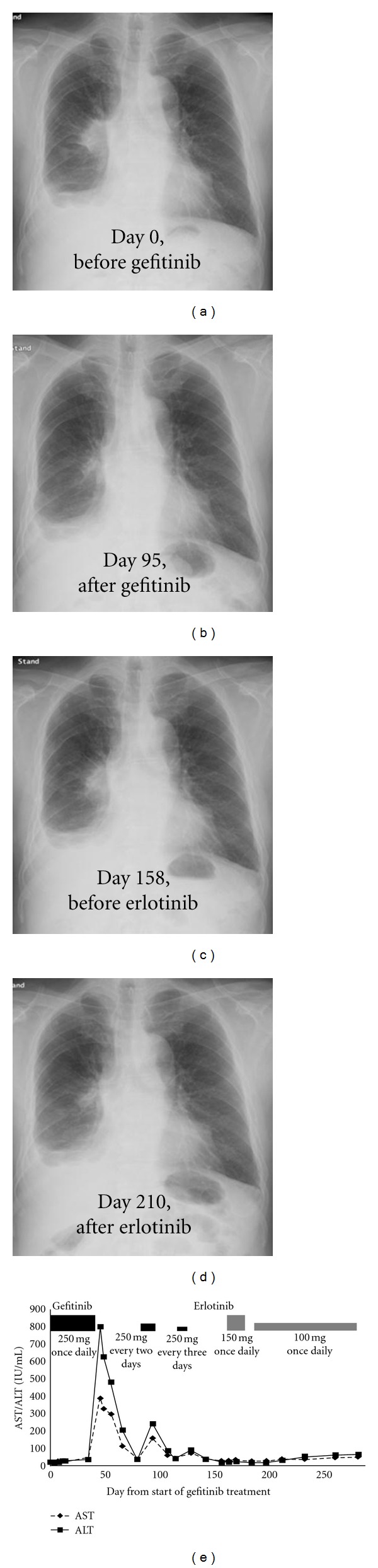
The course of chest X-ray images and transaminase levels during gefitinib and erlotinib treatments.

After 6 weeks of gefitinib treatment, the patient developed substantially elevated hepatic enzymes (AST 386 IU/L, range, 13–31; ALT 801 IU/L, range, 8–34), and the gefitinib treatment was discontinued. A chest X-ray demonstrated mass opacity at the right hilar area, and a computed tomography (CT) scan of the chest and the abdomen showed mass opacity involving the right hilar lymph node without any signs of liver metastasis (Figure 1). A baseline blood test revealed that the electrolyte levels and renal function were normal. The number of eosinophils, which increase in allergic reaction, was also within normal limit. The patient had no previous history of liver disease, excess alcohol intake, hepatitis, or liver metastasis. The patient did have a history of diabetes mellitus that was controlled by oral medication and prostate cancer that was well controlled by periodic hormonal therapy with leuprorelin. He was not routinely prescribed a medicine other than these medications. In addition, he did not take any supplement. Gefitinib treatment was reintroduced with an intermittent schedule after the patient's transaminase levels normalized; however, the enzyme levels rose again when 250 mg of gefitinib was administered every two days (Figure 2). The lung cancer progressed after two weeks of treatment with 250 mg of gefitinib administered every three days. Therefore, the treatment was switched to erlotinib (150 mg once daily) 159 days after the initiation of gefitinib, and the tumor decreased in size, as determined by chest X-ray (Figure 2). Although the patient experienced grade 2 fatigue and nausea, as assessed by CTCAE ver. 3.0, he recovered after the treatment was suspended and then reinitiated at a reduced dose of 100 mg once daily. Tumor control and normal levels of liver enzymes were maintained, but the tumor gradually grew larger 7 weeks after the administration of erlotinib. Erlotinib was subsequently discontinued, and chemotherapy with pemetrexed (500 mg/m^2^) was administered. We had stable disease during pemetrexed treatment. The patient then received palliative care combined with steroids, and he survived for 24 months after the initiation of erlotinib therapy. The patient died as a result of complications from the malignancy in May 2011.

## 3. Discussion

The management of gefitinib-related hepatotoxicity provides great value to patients, especially those patients with lung adenocarcinoma harboring an EGFR mutation who are expected to respond to EGFR-TKI. There are two strategies to overcome gefitinib-related hepatotoxicity: (1) an intermittent schedule of gefitinib administration (once every 2 or 5 days) and (2) the use of erlotinib instead of gefitinib. Both strategies were used in the treatment of the patient reported herein.

The reintroduction of gefitinib administration at the same dose after its discontinuation due to hepatotoxicity has been reported in two cases and resulted in the discontinuation of gefitinib treatment because of the repeated elevation of serum transaminase levels [12, 13]. Tomisaki et al. described a case in which gefitinib was reintroduction at an intermittent schedule of every two days; this intermittent schedule resulted in successful tumor control and reduced toxicity, such as grade 3 skin trouble and rash [14]. This alternate-day regimen is in widespread clinical use to control the complications of gefitinib, although it is still unknown whether this schedule confers a survival benefit. Seki et al. reported that the intermittent administration of gefitinib every 5 days yielded successful tumor control for 8 weeks and reduced hepatotoxicity [15]. This strategy for intermittent gefitinib dosing was based on data from the IDEAL 1 trial, in which grade 1 to 4 elevations of transaminases were more common in patients receiving a dose of 500 mg/day (24%) than in patients receiving 250 mg/day (13%) [3]. In addition, the maximum plasma concentration (*C*_max⁡_) and the AUC were dependent on the number of consecutive days gefitinib was administered in two phase 1 trials [9, 10]; that is, 14 consecutive days dosing resulted in the *C*_max⁡_ and the AUC 2.3-fold and 4.0-fold higher than a single dose, respectively, [9]. In our case, we first reintroduced gefitinib with an intermittent treatment schedule of every two days and then every three days; however, unlike the above-mentioned cases, gefitinib did not yield reduced hepatotoxicity when administered every two days and it did not yield tumor control when administered every three days. Therefore, we changed the medication from gefitinib to erlotinib.

Although gefitinib and erlotinib are similar anilinoquinazoline EGFR-TKIs, the incidence of grade 3/4 liver transaminase levels was reported to be higher with gefitinib than with erlotinib in three phase 3 studies [16–18]. One of the reasons why liver dysfunction is more frequent in gefitinib group than in erlotinib group is thought to be the difference between their metabolisms. Although they undergo extensive metabolism, mainly via hepatic and intestinal cytochrome P450 (CYP) 3A4, gefitinib is also metabolized via CYP 2D6, whereas erlotinib is also metabolized via CYP 1A2 [19]. Therefore, the deletion or inactivation in the gene encoding CYP2D6 might have caused gefitinib-related hepatotoxicity. Furthermore, the standard dose of gefitinib (250 mg/day) is lower than the maximal tolerated dose (MTD); however, the approved daily dose of erlotinib (150 mg/day) is equal to the MTD, because response and survival were not different between 250 and 500 mg of gefitinib among two phase 2 trials [3, 20]. Thus, an erlotinib dosage close to the MTD is considered to achieve more effective drug concentrations when compared with gefitinib administered at an intermittent schedule. That is why this change in medication worked well for our patient, in terms of both reduced toxicity and successful tumor control. In fact, successful treatment with erlotinib after gefitinib-related severe hepatotoxicity has been reported by Takeda et al. [21], although an intermittent schedule of gefitinib was not used in their case (unlike that in our case).

In conclusion, the present case illustrates helpful treatment with erlotinib for a patient with gefitinib-related hepatotoxicity and lung adenocarcinoma refractory to an intermittent schedule of administration. Erlotinib can be an effective and well-tolerated treatment option for patients for whom gefitinib has been discontinued because of severe hepatotoxicity irrespective of their response to gefitinib.
